# Reliability of Concentric, Eccentric and Isometric Knee Extension and Flexion when using the REV9000 Isokinetic Dynamometer

**DOI:** 10.2478/hukin-2013-0024

**Published:** 2013-07-05

**Authors:** Alberto César Pereira de Carvalho Froufe Andrade, Paolo Caserotti, Carlos Manuel Pereira de Carvalho, Eduardo André de Azevedo Abade, António Jaime da Eira Sampaio

**Affiliations:** 1 Research Center of Sports Science, Health and Human Development (CIDESD).; 2 High Institute of Maia (ISMAI), Portugal.; 3 University of Trás-os-Montes e Alto Douro (UTAD), Portugal.; 4 University of Southern Denmark, Institute of Sports Science and Clinical Biomechanics.

**Keywords:** reproducibility, quadriceps, hamstring, peak torque, imbalance ratios, isokinetics

## Abstract

The aim of this study was to assess the reliability of isokinetic and ISO knee extensor and flexor muscle strength when using the REV9000 (Technogym) isokinetic dynamometer. Moreover, the reliability of several strength imbalance indices and bilateral ratios were also examined. Twenty-four physically active healthy subjects (age 23±3 years) underwent three testing sessions, two on the same day and a third, 7 days later. All sessions proceeded in the same order: five concentric contractions at 60ºs-1 followed by an isometric contraction (5 seconds) and five eccentric contractions (60ºs-1). The results of this study showed a high reproducibility in eccentric (0.95–0.97), concentric (0.95–0.96) and isometric (0.93–0.96), isokinetic strength for knee extensor and flexor muscles, thus indicating that the REV9000 isokinetic dynamometer can be used in future sports performance studies. A low-to-moderate reliability was found in the isokinetic strength bilateral ratios while the Hamstring:Quadricep concentric ratio showed moderate reliability. The highest reliability (>0.90) was observed in the dynamic control ratio (Hamstring eccentric:Quadricep concentric) which consequently confirms that it is a more valid indicator for imbalanced reciprocal parameters and can be used in rehabilitation and sports medicine.

## Introduction

The use of isokinetic dynamometers to assess muscle function has become progressively popular in sport, research and clinical settings. Isokinetic devices assess joint and muscle maximal concentric (CON), eccentric (ECC) and isometric (ISO) strength under constant velocities throughout the whole range of motion. Several studies have used isokinetic dynamometers to assess ISO and dynamic (CON and ECC) strength of the knee extensor and flexor muscles ( Bardis et al., 2004 ; Cotte and Ferret, 2003 ). Isokinetic devices are not only used in rehabilitation and sports medicine to assess risk factors such as low strength muscles but also in developing rehabilitation programmes for knee and hamstring injuries or imbalances ( Croisier, 2004 ; Dauty et al., 2003 ; Impellizzeri et al., 2008 ; Kannus, 1994 ). Additionally, some authors have suggested the use of preseason screening of unilateral and bilateral strength imbalance in healthy subjects so as to identify the athletes at a high risk of incurring lower limb injuries during training or competition ( Croisier, 2004 ). It seems consensual that reliable measures are essential to improve the accuracy of ISO, ECC and CON strength evaluations. There are numerous factors that can influence the measurement of reproducibility, such as measuring devices, testing procedures and the individuals’ unique characteristics.

Some studies have previously evaluated the reliability of isokinetic devices such as Biodex ( Feiring et al., 1990 ; Lund et al., 2005 ; Ordway et al., 2006 ), Cybex ( Bandy and Mclaughlin, 1993 ; Blacker et al., 2010 ; Impellizzeri et al., 2008 ; Li et al., 1996 ), Kin Com ( Sole et al., 2007 ; Tredinnick and Duncan, 1988 ), Con-Trex ( Bardis et al., 2004 ; Maffiuletti et al., 2007 ), Merac ( Capranica et al., 1998 ), Lido ( Iga et al., 2006 ) and iSAM 9000 ( Orri and Darden, 2008 ). Nevertheless, no study has assessed the reliability of CON, ISO and ECC by using REV9000 (Technogym). In addition, no information concerning the reliability of lower limb strength imbalance indices using REV9000 are available and very few studies have examined the reliability of such when using Cybex ( Sole et al., 2007 ) and Kin Com ( Impellizzeri et al., 2008 ) in spite of their widespread use.

Thus, this study used the REV9000 isokinetic device (Technogym) on a group of healthy individuals and examined the reliability of the peak torque of the knee extensor and flexor muscles. In addition, it examined the reliability of commonly used strength imbalance indices, such as concentric hamstring (Hcon)/concentric quadriceps (Qcon); eccentric quadriceps (Qecc)/concentric quadriceps (Qcon); eccentric hamstring (Hecc)/concentric hamstring (Hcon); eccentric hamstring (Hecc)/concentric quadriceps (Qcon) and bilateral CON, ISO and ECC ratios. These assessment aims have never been studied with the use of a REV9000 dynamometer.

## Material and Methods

### Procedures

All sessions were carried out at the same time (4p.m.), approximately 3 hours after lunch. They were preceded by a 10 minute warm-up on an ergocycle (70rpm at 50 W) and stretching exercises (for hamstrings and quadriceps) where subjects sat with their thighs at an angle of 85º to the trunk. The mechanical axis of the dynamometer was aligned with the lateral epicondyle of the knee. The trunk and thighs were stabilized with belts. The knee range of motion was 70º (20 to 90º of flexion); the lever arm was positioned at the distal third of the leg; torque was gravity-corrected and dynamometer calibration was performed before every session in accordance with the manufacturers’ instructions. All sessions began with the assessment of the right lower limb and proceeded in the same order: 5 CON contractions at 60ºs ^−1^ followed by ISO muscle contractions (flexion and extension) during 5 seconds (at 60º of flexion) and 5 ECC contractions at 60ºs ^−1^ .

A 90-second recovery period was allowed between CON, ISO and ECC tests. All subjects benefited from both visual feedback and verbal encouragement. The highest value for each testing condition was considered for statistical analysis. All procedures to assess muscular strength and power ( Brown and Weir, 2001 ) were based on the recommendations of the American Society of Exercise Physiologists. All tests were performed with the use of the REV9000 (Technogym, Italy) isokinetic dynamometer and controlled by the same qualified technician.

The strength difference between the hamstrings and quadriceps of the same limb (unilateral) was calculated by the ratio between the peak torque produced concentrically during the isokinetic tests (Hcon:Qcon). The dynamic control ratio was calculated by the ratio between the peak torque produced eccentrically by the hamstrings (Hecc), and concentrically by the quadriceps (Qcon) ( Impellizzeri et al., 2008 ). This variable (Hecc:Qcon) is an indicator of the extent in which hamstrings are capable of counteracting the anterior tibial shear induced by maximal quadriceps muscles ( Aagaard et al., 1995 ). In fact, ECC antagonist/CON agonist moment ratio has already been suggested to be a more valid indicator of muscular imbalance than the eccentric (ECC/ECC) or concentric (CON/CON) reciprocal parameters ( Kellis and Baltzopoulos, 1995 ).

Previous research has already introduced different ways to calculate the bilateral lower limb strength asymmetry ( Keays et al., 2003 ; Kuruganti and Seaman, 2006 ). The present study used a right/left comparison, this is, an absolute strength asymmetry of 40 Nm in a subject with a stronger right leg (right leg = 200 Nm; left leg = 160 Nm) would correspond to 1.25, however, if the same subject performed stronger with the left leg the ratio would be 0.80 ( Impellizzeri et al., 2008 ).

### Analysis

All strength imbalance indices and peak torque data were calculated with mean and standard deviation. Reliability concerned the degree in which individuals maintained their position in a sample involving repeated measurements ( Atkinson and Nevill, 1998 ). This type of reliability was assessed with the intraclass correlation coefficient (ICC). ICC above 0.90 was considered high, between 0.80 and 0.90 as moderate and below 0.80 as low ( Stuchlikova, 1995 ).

Detection of systematic biases was performed by using repeated ANOVA measurements while post-hoc differences were assessed by using the Bonferroni test ( Atkinson and Nevill, 1998 ). Plotting the residual versus predicted values and calculating the Pearson`s correlation coefficient with significant correlations indicated and examined heteroscedasticity, i.e. error depending on the magnitude of the mean.

Standard error of measurement (SEM) reflects the degree of an individuals’ test score variation that might be expected from measurement error ( Sleivert and Wenger, 1994 ) and in a case of a perfect agreement is equal to zero. This within-subject standard deviation is not only known as the typical error in a measurement but it also expresses the band of confidence around an individual’s raw score. The most common way to calculate this statistic is by means of the following equation: *SEM* = *SD ×* ( *1* − *ICC* ) ^0.5^ , where SD is the sample standard deviation and ICC is the calculated intraclass correlation coefficient ( Atkinson and Nevill, 1998 ). The use of SD in the equation, in effect, partially “cancels out” the inter-individual variation that was used to calculate the ICC ( Anthony, 1999 ). The comparison across the sample, measurement error was also expressed as the *SEM%* [SEM% = (SEM/mean) × 100] to produce a unitless indicator of error magnitude.

The minimum detectable change (MDC) defined as 95% limits of agreement ( Atkinson and Nevill, 1998 ; Weir, 2005 ), is also called the smallest real difference (SRD) and defined as 95% confidence limit of the standard error of measurement (SEM). This threshold is required to detect statistically significant change in an individual when taking into account the variability associated with both the measurement technique and experimental sample ( Beckerman et al., 2001 ; Beckerman et al., 1996 ). The SRD was calculated for each condition by using the equation *SRD or MDC* = *1.96* × *2* ^0.5^ × *SEM.*

The probability of type I error (alpha) was set at priori at 0.05 in all statistical analysis.

All procedures were performed with SPSS 17.0 statistical software (SPSS Inc., Chicago, IL, USA).

## Results

Table 1 presents the mean ± SD and ICC of the quadriceps peak torque. High reliability (>0.90) was identified for the contraction mode. The highest and lowest ICC for peak torque was found in the eccentric contraction for the left knee flexor (0.97) and in ISO contraction of the left knee extensor (0.93), respectively. For concentric and eccentric contractions, the ICC was high in the interval of 0.95 to 0.97 (high reliability). Moreover, the ICC of ISO contractions had high reliability with values ranging between 0.93 and 0.96. In what concerns the ICC of peak torque on the three attempts, all isokinetic and ISO strength outcomes showed high reliability for both extensor and flexor knee muscles. No significant differences were found between all sessions.

Table 2 presents the mean ± SD and ICC of unilateral and bilateral strength imbalance ratios obtained in the three trials. ICC values for Hcon:Qcon and Hecc:Qecc were moderate-to-high. Similarly, all ICC values for Hecc:Hcon and Qecc:Qcon showed moderate reliability. In regards to Hecc:Qcon ratio, the reliability was high for both right and left lower limbs. A significant effect of time was found for Qecc:Qcon ratio, which was lower in the first trial compared to the second and third trials. This effect was not found in the other unilateral ratios. The reliability of bilateral strength imbalance ratios obtained in the three trials was low-to-moderate. When compared to the bilateral hamstring ratio values (0.63–0.73), the ICC values for bilateral quadriceps ratios (0.71–0.81) were higher.

## Discussion

The results of this study showed a high reliability of CON, ECC and ISO muscle strength assessment by using the REV9000 dynamometer for both knee extensor and flexor muscles. When comparing the results in the same session and between sessions, both reveal high reliability. However, ICC values found in the same session were always higher.

## Discussion

When analysing the reliability among all sessions, the highest ICC for peak torque was found in the ECC contraction for the left knee flexor (0.97) and the lowest for the ICC in ISO contraction of the left knee extensor (0.93). For CON and ECC contractions, the ICC interval was 0.95 – 0.97 and 0.93 – 0.96 for ISO contractions.

These results were identical to a previous study ( Maffiuletti et al., 2007 ) which showed values of 0.97 (Quad)/0.98(Ham) for concentric contraction at 60ºs-1 and 0.97 (Quad)/0.97(Ham) for ISO contractions. Another study ( Impellizzeri et al., 2008 ) found similar ICC results of 0.95(Quad)/0.98(Ham) for the right lower limb and 0.95(Quad)/0.93(Ham) for the left lower limb in concentric contractions at 60ºs-1. In what relates to eccentric contractions (60ºs-1), the same study had values of 0.96(Quad)/0.94(Ham) for the right lower limb and 0.95(Quad)/0.97(Ham) for left lower limb.

The present study showed that the right knee flexor reliability peak torque was slightly higher than the knee extensor, which is not in accordance with previous findings ( Impellizzeri et al., 2008 ; Li et al.,1996 ; Maffiuletti et al., 2007 ). Nevertheless, since the magnitude of the differences in ICC was very low, no parameter showed clear superior reliability when compared to the others.

The SEM percentual values of the absolute isokinetic strength measures ranged from 3.6% to 5.7% and the MDC from 9.7% to 15.8%. These results were similar to a previous study that used a Biodex isokinetic device, which found SEM values of about 7% for knee extensors and 9% for knee flexors, with corresponding MDC ranging from 13% to 17% ( Lund et al., 2005 ). SEM values ranging from 4% to 7% and MDC ranging from 10% to 20% with a Cybex 6000 Dauty & Rochcongar ( Dauty and Rochcongar, 2001 ) and SEM values ranging from 4.3% to 6.7% and MDC from 11.1% to 19% ( Impellizzeri et al., 2008 ) have already been reported. Nevertheless, a previous study has found higher SEM and MDC values, however the assessed population was based on individuals with mild and moderate osteoarthritis of the knee and not on healthy subjects ( Germanou et al., 2007 ).

The present study recognized the reliability of most common indices of strength imbalance ratios by using the REV9000. For bilateral quadriceps ratios, the ICC results were 0.81 for CON (at 60ºs-1), 0.85 for ISO and 0.71 for ECC contraction (at 60ºs-1). For bilateral hamstring ratios, ICC values were 0.63, 0.73 and 0.66 for CON, ISO and ECC contractions, respectively. These ICC values were slightly higher than those previously reported ( Impellizzeri et al., 2008 ): 0.69 and 0.67, for the bilateral quadriceps ratio, using the CON and ECC peak torque at 60ºs-1. For bilateral hamstring ratios, the ICC values were 0.59 using the CON peak torque and 0.69 for ECC peak torque (at 60ºs-1). Another study showed even lower ICC values 0.42 at 30ºs-1 and 0.81 at 90ºs-1 for the bilateral quadriceps ratio ( Hsu et al., 2002 ). However, one of the main reasons for these different results may be related to the participation of nine stroke patients.

Relatively to the Hcon:Qcon ratio, ICC values ranging from 0.36 to 0.93 in 10 healthy men after one session of familiarization had already been reported ( Gleeson and Mercer, 1992 ). Another study found ICC values of 0.79 and 0.65 for the unilateral hamstring-to-quadriceps ratio using the CON peak torque (at 60ºs-1) of the right and left lower limbs ( Impellizzeri et al., 2008 ). Moreover, ICC values of 0.43 at 60ºs-1 were already found for the unilateral hamstring-to-quadriceps ratio using the CON peak torque of the dominant lower limb ( Sole et al., 2007 ). All these ICC results are considered low, however the present study found a moderate ICC of Hcon:Qcon ratio at 60ºs-1 (0.89 for the right leg and 0.87 for the left leg) and a high ICC for Hecc:Qecc ratio also at 60ºs-1 (0.91 for the right leg and 0.92 for the left leg). All together, these studies seem to show moderate reliability of these imbalance ratios, as previously suggested ( Dauty et al., 2003 ; Gleeson and Mercer, 1992 ; Impellizzeri et al., 2008 ).

The present study provided higher ICC values for the Hecc:Qcon ratio measured at 60ºs-1 (0.92 for the right leg and 0.90 for the left leg) when compared to Hcon:Qcon ratios. These results are also higher than those found in previous reports. Moderate ICC values (0.87 for the right leg and 0.80 for the left leg) were already found for the Hecc:Qcon ratio measured at 60ºs-1 ( Impellizzeri et al., 2008 ) and low ICC values (0.73 in dominant leg) were also reported for the Hecc/Qcon ratio at 60ºs-1 ( Sole et al., 2007 ).

Subsequently, similar reliability values of knee extension and flexion measurements were found when comparing REV9000 with Cybex NORM and Con-Trex machines. Apparently, there were no differences in the results of reliability across all these devices; however there was a higher ICC with the REV9000 isokinetic dynamometer compared to the Cybex NORM and Con-Trex. In fact, it seems that reliability could present higher values if the subjects were more familiarized with the isokinetic tests because of the inherent learning effects.

In conclusion, the present study showed a high reproducibility of ECC, CON and ISO isokinetic strength for knee extensor and flexor muscles when using REV9000. As a consequence, it seems that this isokinetic device can be used for future sports performance studies and to improve the training process. The high reproducibility when using REV9000 may help coaches and clinicians to evaluate with accuracy the isometric and dynamic strength of the knee extensors and flexors, accelerate strength gains during rehabilitation processes and prevent possible risk factors of knee and hamstring injury or imbalances. A low-to-moderate reliability for the isokinetic strength bilateral ratios and a moderate reliability of Hcon:Qcon ratio was found. Additionally, high ICC for dynamic control ratio Hecc:Qcon and Hecc:Qecc ratio measured at 60ºs-1 was found, indicating that these ratios can be used in rehabilitation and sports medicine.

## Figures and Tables

**Table 1:** *
Mean, Standard Deviation, ICC intraclass correlation coefficient and CI confidence interval of PT (Nm) for knee extensor and flexor muscles obtained during isokinetic tests.
*

Peak torque (Nm)	** Mean ± SD **			Within	Between	All	P-value	95% CI	SEM (%) ±
		
	Session 1	Session 2	Session 3	ICC (2–3)	ICC (1–2)	ICC		lower; upper	MDC (%)
*** Quadriceps right ***									
Concentric at 60ºs ^−1^	229.4 ± 37.5	223.5 ± 33.9	224.5 ± 37.5	.93	.93	.95	0.290	0.90; 0.98	3.6 ± 9.9
Isometric	257.8 ± 46.9	255.3 ± 55.7	245.9 ± 55.4	.99	.92	.96	0.210	0.92; 0.98	4.2 ± 11.6
Eccentric at 60ºs ^−1^	303.0 ± 63.6	303.3 ± 66.7	298.4 ± 62.7	.97	.93	.96	0.055	0.93; 0.98	4.1 ± 11.3
*** Hamstrings right ***									
Concentric at 60ºs ^−1^	125.0 ± 27.3	129.9 ± 29.6	129.7 ± 27.9	.97	.89	.95	0.021	0.90; 0.98	4.9 ± 13.5
Isometric	138.1 ± 34.1	139.9 ± 33.8	131.1 ± 33.5	.99	.93	.96	0.660	0.92; 0.98	4.8 ± 13.3
Eccentric at 60ºs ^−1^	171.5 ± 33.8	166.9 ± 40.9	162.4 ± 40.3	.97	.91	.95	0.088	0.91; 0.98	5.1 ± 14.1
*** Quadriceps left ***									
Concentric at 60ºs ^−1^	219.4 ± 36.5	215.9 ± 38.0	213.8 ± 38.5	.96	.96	.95	0.343	0.91; 0.98	3.8 ± 10.5
Isometric	236.0 ± 43.9	235.3 ± 46.5	235.3 ± 43.7	.94	.94	.93	0.688	0.87; 0.97	4.8 ± 13.3
Eccentric at 60ºs ^−1^	267.8 ± 58.8	268.4 ± 59.9	271.8 ± 59.6	.96	.96	.96	0.988	0.92; 0.98	4.4 ± 12.2
*** Hamstrings left ***									
Concentric at 60ºs ^−1^	129.3 ± 26.6	127.6 ± 25.9	130.0 ± 31.2	.96	.96	.96	0.809	0.91; 0.98	4.8 ± 13.3
Isometric	129.8 ± 33.5	129.6 ± 31.2	127.7 ± 31.0	.91	.91	.94	0.755	0.89; 0.97	5.7 ± 15.8
Eccentric at 60ºs ^−1^	162.2 ± 36.4	156.2 ± 33.7	161.8 ± 34.1	.98	.98	.97	0.075	0.94; 0.99	3.5 ± 9.7

**Table 2:** *
Mean, Standard Deviation, ICC intraclass correlation coefficient and CI confidence interval of the lower limb strength imbalance indices.
*

Parameters	** Mean ± SD **			P-value	ICC	95% CI	SEM (%) ±
		
	Session 1	Session 2	Session 3			lower; upper	MDC (%)
*** Unilateral hamstring quadriceps ratio right ***							
Concentric at 60ºs ^−1^	0.55 ± 0.08	0.58 ± 0.09	0.58 ± 0.08	0.012	.89	0.77; 0.95	4.7 ± 13.0
Isometric	0. 54 ± 0.11	0.58 ± 0.23	0.56 ± 0.21	0.496	.87	0.74; 0.94	13.8 ± 38.2
Eccentric at 60ºs ^−1^	0.58 ± 0.11	0.56 ± 0.14	0.55 ± 0.11	0.326	.91	0.82; 0.96	5.6 ± 15.5
Qecc:Qconc at 60ºs ^−1^	1.33 ± 0.22	1.35 ± 0.15	1.34 ± 0.28	0.790	.85	0.70; 0.93	8.1 ± 22.4
Hecc:Hconc at 60ºs ^−1^	1.39 ± 0.19	1.29 ± 0.17	1.26 ± 0.22	0.008	.72	0.45; 0.87	8.3 ± 22.9
Hecc:Qconc at 60ºs ^−1^	0.76 ± 0.14	0.75 ± 0.17	0.73 ± 0.19	0.519	.92	0.84; 0.96	6.9 ± 19.1
*** Unilateral hamstring quadriceps ratio left ***							
Concentric at 60ºs ^−1^	0.59 ± 0.09	0.60 ± 0.09	0.61 ± 0.10	0.319	.87	0.75; 0.94	6.1 ± 16.8
Isometric	0. 56 ± 0.12	0.56 ± 0.13	0.55 ± 0.14	0.953	.90	0.79; 0.95	7.8 ± 21.6
Eccentric at 60ºs ^−1^	0.62 ± 0.14	0.60 ± 0.13	0.61 ± 0.10	0.442	.91	0.82; 0.96	4.8 ± 13.2
Qecc:Qconc at 60ºs ^−1^	1.22 ± 0.17	1.25 ± 0.18	1.27 ± 0.17	0.199	.86	0.73; 0.94	5.1 ± 14.1
Hecc:Hconc at 60ºs ^−1^	1.26 ± 0.18	1.23 ± 0.14	1.26 ± 0.18	0.551	.71	0.43; 0.87	7.6 ± 21.0
Hecc:Qconc at 60ºs ^−1^	0.74 ± 0.11	0.73 ± 0.12	0.76 ± 0.11	0.115	.90	0.80; 0.95	4.5 ± 12.4
*** Bilateral quadriceps ratio ***							
Concentric at 60ºs ^−1^	0.96 ± 0.12	0.97 ± 0.13	0.96 ± 0.15	0.941	.81	0.63; 0.91	6.7 ± 18.5
Isometric	0. 92 ± 0.13	0.94 ± 0.18	0.98 ± 0.18	0.137	.85	0.71; 0.93	7.5 ± 20.7
Eccentric at 60ºs ^−1^	0.89 ± 0.10	0.89 ± 0.13	0.92 ± 0.13	0.463	.71	0.43; 0.87	7.8 ± 21.6
*** Bilateral hamstring ratio ***							
Concentric at 60ºs ^−1^	1.05 ± 0.17	0.99 ± 0.12	1.01 ± 0.14	0.221	.63	0.26; 0.83	8.0 ± 22.1
Isometric	0. 95 ± 0.14	0.94 ± 0.18	1.00 ± 0.20	0.260	.73	0.47; 0.88	10.8 ± 29.9
Eccentric at 60ºs ^−1^	0.95 ± 0.13	0.95 ± 0.13	1.01 ± 0.15	0.070	.66	0.33; 0.84	9.1 ± 25.2
